# Identification and characterization of proteins, lipids, and metabolites in two organic fertilizer products derived from different nutrient sources

**DOI:** 10.1186/s13765-021-00625-2

**Published:** 2021-10-05

**Authors:** Jianyu Li, Xin Zhao, Laura S. Bailey, Manasi N. Kamat, Kari B. Basso

**Affiliations:** 1 Horticultural Sciences Department, University of Florida, Gainesville, FL 32611-0690 USA; 2 Department of Chemistry, University of Florida, Gainesville, FL 32611-7200 USA

**Keywords:** Biochemical composition, LC–MS/MS, Liquid chromatography–tandem mass spectrometry, Lipidomics, Metabolomics, Nutrient availability, Organic nutrient source, Proteomics

## Abstract

The biochemical composition of organic fertilizers largely determines their nutrient supply characteristics following soil application as well as their potential impact on soil microbial communities. Yet, limited information is available regarding the biochemical composition of organic fertilizers derived from different nutrient sources. Here, we qualitatively analyzed the presence and abundance of proteins, lipids, and metabolites in a liquid fish fertilizer (LFF) product and a type of granular organic fertilizer (GOF) commonly used in organic vegetable production, using liquid chromatography–tandem mass spectrometry (LC–MS/MS). Our results suggest that the presence and abundance of proteins, lipids, and metabolites differ greatly between GOF and LFF. The qualitative analysis shows LFF as a rich source of metabolites, while complex proteins and long-chain saturated fatty acids are dominant in GOF. The degree of biochemical composition complexity may help explain the varying impacts of different types of organic fertilizers on nutrient availability, soil health, and environmental quality.

**Supplementary Information:**

The online version contains supplementary material available at 10.1186/s13765-021-00625-2.

## Introduction

Organic fertilizers derived from various animal and plant-based byproducts have been widely used as nutrient sources for organic vegetable crop production. Compared to synthetic chemical fertilizers, organic fertilizers tend to have lower nutrient content along with variability in composition that depends on ingredients. However, organic fertilizers often contain beneficial microorganisms and are generally rich in organic carbon (C) [1]. Applications of organic fertilizers have been shown to increase soil organic matter content, enhance overall soil enzyme activity [2], and influence microbial community composition through addition of C and nitrogen (N)-rich organic compounds [3–6]. At the same time, the nutrient release process of organic fertilizers relies substantially on the complex biochemical transformation activities mediated by soil microbes [7] in addition to environmental conditions.

Early studies indicated that the general C/N ratio and nutrient analysis of organic fertilizers might not offer sufficient information for predicting nutrient release patterns, while the biochemical composition of organic fertilizers could be highly associated with mineralization kinetics [8, 9]. Numerous methods have been developed to estimate N availability in soils after organic fertilizer application, such as incubation-based N mineralization studies [10, 11]. Most recently, the extractable soil protein pool was suggested to be a soil health indicator of potentially available organic N [12]. Plant metabolite compounds also influence soil nutrient cycling, such as C and N mineralization, through their impact on soil organisms [13]. The input of lipids, especially the long-chain compounds, play an important role in soil health enhancement including the accumulation of soil organic matter content [14]. Liquid chromatography–tandem mass spectrometry (LC–MS/MS) with high-throughput capacities has been demonstrated to be a sensitive and powerful analytical tool for detecting proteins, lipids, and other metabolites from biological materials of various sources including soil [15] and plant tissue samples [16–18]. However, limited information is available regarding specific biochemical compounds contained in organic fertilizers that may affect soil microbial communities and nutrient availability as well as environmental quality. LC–MS/MS has rarely been used to compare biochemical compositions of organic fertilizers with different nutrient sources. Here, we chose to examine two commercially available organic fertilizer products, a liquid fish fertilizer (LFF) derived from enzymatically digested fish proteins and a granular organic fertilizer (GOF) containing feather meal and other animal waste materials, which represent commonly used nutrient sources for organic crop production [19].

Therefore, the objective of this pilot study was to employ LC–MS/MS to identify and characterize proteins, lipids, and metabolites in two commonly used commercial organic fertilizer products for qualitative comparisons of their biochemical characteristics.

## Materials and methods

**Figure 1:**
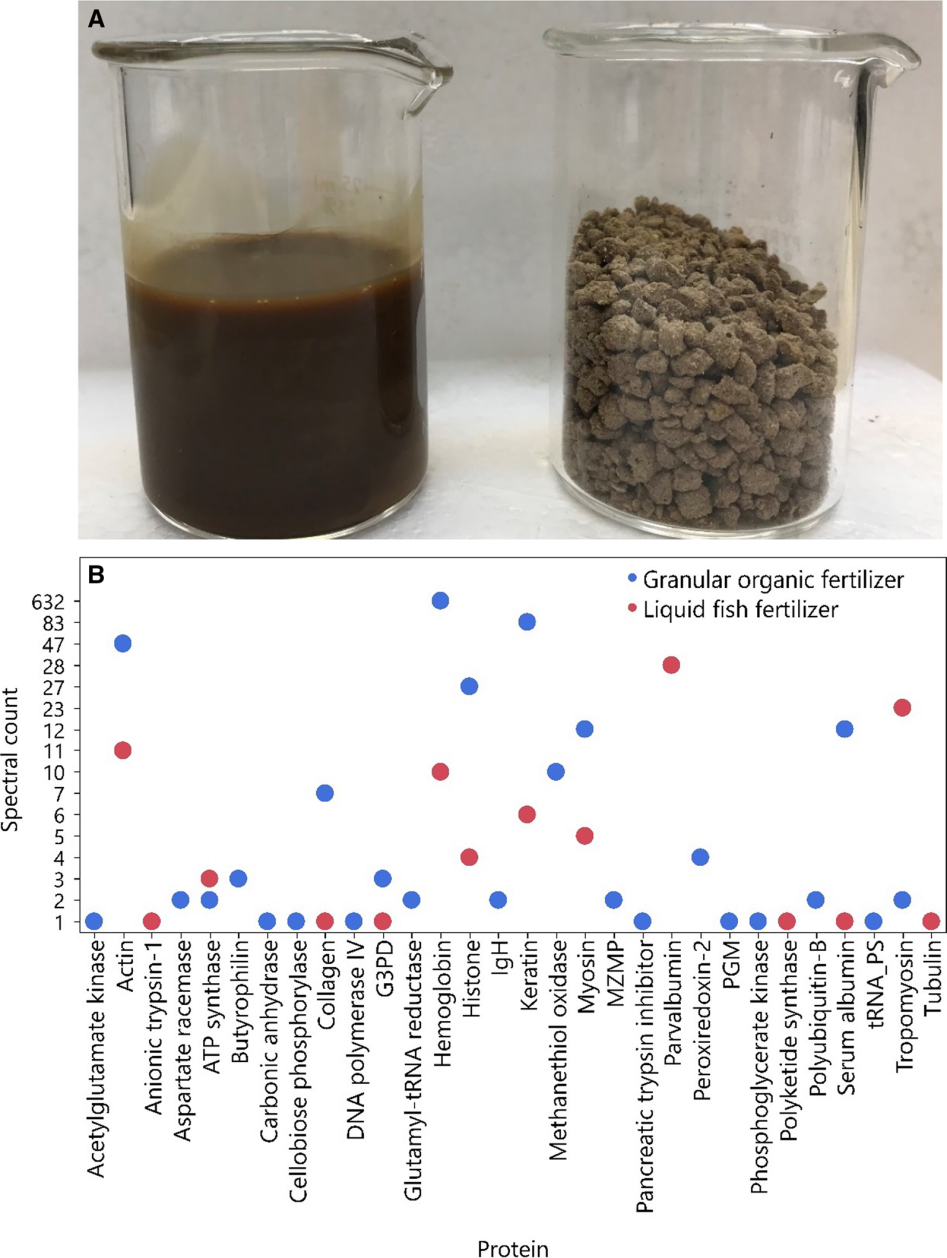
**A** **Visual appearance of the two types of organic fertilizer products analyzed**. Left: liquid fish fertilizer (LFF); Right: granular organic fertilizer (GOF). **B** **Total spectral counts for each protein identified in GOF and LFF**. G3PD: glyceraldehyde-3-phosphate dehydrogenase, IgH: immunoglobulin heavy chain, MZMP: mitochondrial zinc maintenance protein, PGM: phosphoglucosamine mutase, tRNA_PS: tRNA pseudouridine synthase. Y-axis scale is made disproportionally to the spectral count in order to capture the wide range of spectral counts of all proteins identified and indicate the actual values of spectral counts for each protein.

The LFF analyzed in this study can be used in organic fertigation systems through drip irrigation, while the GOF may be used for preplant application and/or sidedressing during the crop production season. Both organic fertilizer products have higher levels of N than phosphorus (P) and potassium (K) (Figure 1A; Additional file 1: Table S1). Equal amounts of LFF (100 µL) and GOF (100 mg) were individually extracted for proteins, lipids, and metabolites. The equivalent mass of 100 µL LFF was determined as 97.5 ± 1.0 mg. Lipids were extracted via a modified Folch method [20], metabolites were extracted by ice-cold methanol, and proteins by acetone/methanol precipitation. The protein pellet was reconstituted in 0.2% surfactant enhancer and concentrations were measured via a Qubit Fluorometer (Thermo Fisher Scientific Inc., Waltham, MA, USA). In-gel protein digestion was performed using trypsin as the enzyme (Promega Corporation, Madison, WI, USA). LC analysis for all was performed on a Dionex UltiMate 3000 RSLCnano system (Thermo Fisher Scientific Inc.). Lipid and metabolites were separately injected (5 µL) on an Acclaim PepMap RSLC C18 column (Thermo Fisher Scientific Inc.) using acidified mobile phases of water and acetonitrile for metabolites and water, acetonitrile, and isopropanol for lipids. Proteins (10 µg) were separated on a PepMAP column using acidified water and acetonitrile.

Both the lipid and metabolite compounds were analyzed on a Bruker Impact II QqTOF mass spectrometer (Bruker Daltonics, Billerica, MA, USA) using electrospray ionization operated in positive mode. Proteins were analyzed on a Thermo Scientific Q Exactive HF Orbitrap mass spectrometer equipped with an EASY Nanospray source operated in positive mode. All employed data dependent collisionally-activated dissociation. Proteomics data were analyzed in Proteome Discoverer (version 2.4) using the SEQUEST HT searching algorithm. Lipids and metabolites were analyzed in MetaboScape (version 4.0). Full experimental details are provided in Additional file 1.

## Results and discussion

### Proteomics

Figure 1B is a graphical summary of total spectral counts for each protein detected in  GOF and  LFF products. Hemoglobin (632 spectral counts), keratin (83 spectral counts), and actin (47 spectral counts) were the major proteins detected in GOF (862 spectral counts in total). In contrast, parvalbumin (28 spectral counts), tropomyosin (23 spectral counts), and actin (11 spectral counts) were abundant in LFF (96 spectral counts in total). Degradation of proteins in the soil can be affected by their intrinsic structures including amyloid fibril formation and glycosylation, accessibility to soil microbes or extracellular enzymes, and complex structure formation with other soil organic compounds such as tannins, lignin, and humic substances [21]. For instance, hemoglobin can bind tannins to form protein–polyphenol complex (PPC) that limits N mineralization due to its resistance to decomposition [22]. As a fibrous structural protein, keratin could be highly recalcitrant in response to microbial degradation because of its molecular architecture that involves formation of disulfide bonds, phosphorylation, and glycosylation [23]. The predominance of hemoglobin and keratin found in GOF might imply lower N mineralization rates of GOF relative to LFF.

### Lipidomics

**Figure 2:**
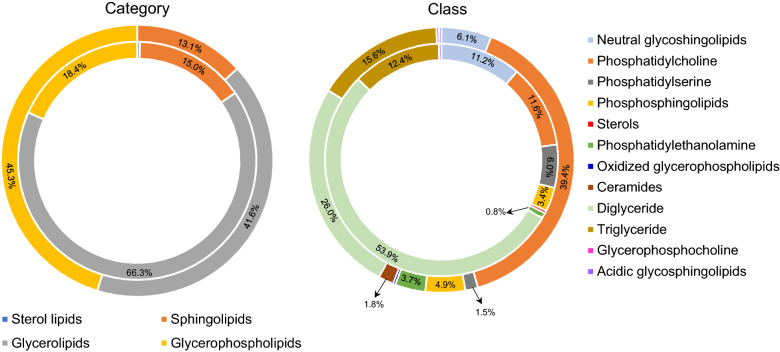
**Different categories and classes of lipids identified in granular organic fertilizer (GOF) and liquid fish fertilizer (LFF)**. The interior donut represents LFF, and the exterior donut represents GOF. The area of each color represents the relative abundance (%) indicated. Based on the Lipid Metabolites and Pathways Strategy (LIPID MAPS) classification system, lipids are divided into four categories: sterol lipids, sphingolipids, glycerolipids, and glycerophospholipids. Each category is further divided into classes. Glycerolipids: diglyceride and triglyceride; Glycerophospholipids: phosphatidylcholine, phosphatidylserine, phosphatidylethanolamine, glycerophosphocholine, and oxidized glycerophospholipids; Sterol lipids: sterols; Sphingolipids: phosphosphingolipids, neutral glycosphingolipids, ceramides, and acidic glycosphingolipids.

Phosphatidylcholine (PC) (e.g., C42, C44, C46) and diglyceride (DAG) (e.g., C31, C35, C37, C39, C41, C43, C47) lipids accounted for 39.4% and 26.0% of the lipids in GOF, respectively, whereas DAGs (e.g., C33, C35, C37, C39, C41, C43, C47) showed a higher classification proportion (53.9%) in LFF (Figure 2). Our data also revealed that the majority of DAGs in both LFF and GOF contained highly polyunsaturated fatty acids (e.g., 30:4, 32:3, 34:5, 40:9, 44:12), which could be more susceptible to microbial degradation in the soil than saturated fatty acid molecules [24]. Conversely, PC lipids containing N and P identified in GOF may degrade and release essential elements rather slowly due to its long-chain structure and the relatively high degree of fatty acid saturation. Overall, the diverse array of lipids identified in organic fertilizers in our study might indicate the potential impact of organic fertilizer application on improving soil health. For instance, lipids may serve as C and energy sources for various lipid-degrading soil microorganisms, such as *Bacillus*, *Arthrobacter*, and *Pseudomonas* [25]. Long-chain lipids (> C20), in particular, are important in C stabilization and humification processes during the accumulation of soil organic matter [26].

### Metabolomics

**Figure 3:**
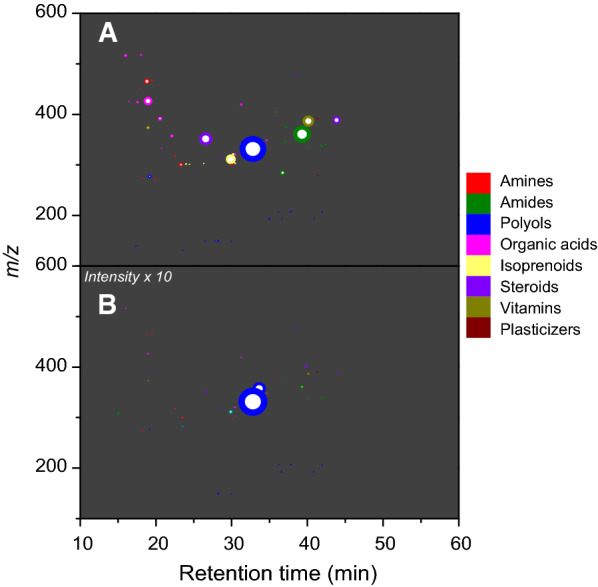
**Survey overview of metabolite presence and relative abundance broken down by class and functionality for liquid fish fertilizer (A) and granular organic fertilizer (B)**. These compounds were annotated and categorized with the help of PubChem based on the general chemical structures of compounds. The compound abundance was determined using the spectral and analyte metabolite matches compiled in MetaboScape. The circle color represents compound category and circle size is proportional to the compound abundance. The abundance values were scaled up by an intensity factor of 10 in plot B in order to make dots visible in plot B.

The identified metabolites (Additional file 1: Table S2) were categorized as amines, amides, polyols, organic acids, steroids, vitamins, isoprenoids, and plasticizers (Figure 3). Comparisons between LFF and GOF suggested that LFF is a richer source of metabolites (Figure 3) and the two organic fertilizers might have different impacts on soil nutrient cycling and soil microbial communities. For example, stearamide (*m/z* 284.2949; retention time (RT) 36.74 min) was detected in LFF but not in GOF. It is interesting that capsaicin (*m/z* 308.2218; RT 15.05 min) was found in GOF but absent in LFF (Additional file 1: Table S2). Although the source of capsaicin is unclear, the GOF that contains capsaicin might potentially demonstrate a deterrent or repellent effect on certain fungi, insects, and mammals due to the irritant property of capsaicin [27]. Four fatty acid amides including pipericine (*m/z* 336.3260; RT 40.04 min), macamide (*m/z* 346.3100; RT 37.08 min), docosanamide (*m/z* 340.3572; RT 42.44 min), and erucamide (*m/z* 338.3416; RT 41.87 min) appeared more abundant in LFF than GOF (Table S2), but statistical analyses with replications were not performed in this exploratory study. These amides may participate in stimulatory activities associated with soil microbial metabolism [28]. Two phenols, including *p*-coumaric acid ethyl ester (*m/z* 193.0861; RT 35.01 min) and gingerol (*m/z* 277.1798; RT 19.20 min), were also abundant in LFF (Table S2). As a product of acidic hydrolysis of *p*-coumaric acid ethyl ester, *p*-coumaric acid can increase soil dehydrogenase activity and abundance of soil bacterial and fungal communities [29, 30]. However, gingerol, another compound abundant in LFF, may display antimicrobial activity [31]. Additionally, given the relatively high acidity (pH = 3.5) and abundant level of organic acids of LFF (Additional file 1: Tables S1, S2), its application could potentially result in a reduction in soil pH and suppression of certain soilborne pathogens in the longer term [32]. The low pH of LFF may be attributed to the abundance of bile acids, such as 3β-hydroxy-5-cholenoic acid (*m/z* 357.2787; RT 22.11 min), 3-oxocholic acid (*m/z* 424.3060; RT 17.59 min), cholic acid (*m/z* 426.3214; RT 18.98 min), and nutriacholic acid (*m/z* 391.2844; RT 20.55 min) (Table S2). Moreover, bacterial degradation products (e.g., androstadienediones) of bile acids could also pose  a potential risk, such as reduced reproduction rates, to invertebrates in agricultural soils [33]. Similarly, as a steroid hormone, progesterone (*m/z* 315.2319; RT 28.67 min), which was discovered in both LFF and GOF (Additional file 1: Table S2), might accumulate in soil and cause adverse impacts on the environment [34].

## Conclusions

Our qualitative analysis is the first study attempting to elucidate the biochemical composition of organic fertilizers, explore its linkage to nutrient availability of organic fertilizers, and envision its impact on soil quality and health. The dominance of complex proteins and long-chain saturated fatty acids contained in GOF suggests that GOF might decompose and release nutrients at a slower rate in the soil relative to LFF. A diverse variety and abundance of metabolites were identified in GOF and LFF, indicating potentially different impacts of these organic fertilizers on soil microbial communities and nutrient availability. Future research can examine more organic fertilizer products derived from various nutrient sources and include quantitative analysis for in-depth comparisons. Overall, our study demonstrates the complexity in biochemical composition of organic fertilizers and suggests the need to further understand how organic fertilizers with different biochemical profiles influence nutrient cycling, soil health, and environmental quality.

## Supplementary Information

**Additional file 1: Table S1. Basic characteristics of the two organic fertilizer products used in this study**.** Table S2. List of metabolites identified by LC-MS/MS in liquid fish fertilizer and granular organic fertilizer**.

## Data Availability

All data generated or analyzed during the present study are included in this published article.

## Abbreviations

LFF: Liquid fish fertilizer
GOF: Granular organic fertilizer
GC/MS: Gas chromatography–mass spectrometry
LC–MS/MS: Liquid chromatography–tandem mass spectrometry
PPC: Protein–polyphenol complex
PC: Phosphatidylcholine
DAG: Diglyceride
