# Supplementary material for: Identification and characterization of proteins, lipids, and metabolites in two organic fertilizer products derived from different nutrient sources
Source: Appl Biol Chem. 2021 Oct 5;64(1):72. doi: 10.1186/s13765-021-00625-2 (PMC8550213; doi:10.1186/s13765-021-00625-2)
Supplement: Supplementary file 1 — Additional file 1: Table S1. Basic characteristics of the two organic fertilizer products used in this study. Table S2. List of metabolites identified by LC-MS/MS in liquid fish fertilizer and granular organic fertilizer. [file 13765_2021_625_MOESM1_ESM.docx]

***Supplementary Information***

**Materials and Methods**

***Materials***

Density measurements of the two organic fertilizer products were used to ensure approximately equal amounts of material were extracted for liquid fish fertilizer (LFF) and granular organic fertilizer (GOF). GOF was hand-ground to a fine powder using a mortar and pestle prior to extraction. Extractions were performed individually for proteins, lipids, and metabolites, using the methods described below. Extracts were then subjected to liquid chromatography-tandem mass spectrometry (LC-MS/MS) analysis.

Ammonium formate (certified crystalline), mobile phase solvents (Optima LC/MS grade), acetone (Optima grade), chloroform (ACS grade), formic acid and trifluoroacetic acid (TFA) (LC/MS grade), dithiothreitol, and iodoacetamide were purchased from Thermo Fisher Scientific Inc. (Waltham, MA, USA). 2,5-di-tert-butyl-4-methylphenol (BHT) was purchased from Aldrich. Ammonium bicarbonate was purchased from MilliporeSigma (St. Louis, MO, USA).

***Lipid preparation***

LFF (100 µL) and GOF (100 mg) were added to 500 µL and 1.2 mL water, respectively, and extracted in 1 mL chloroform and 500 µL methanol with 1 mM BHT. The mixtures were vortexed for 30 sec before incubating at -20 °C for 2 h. The samples were then centrifuged for 5 min at 1,500 rpm. The bottom layer was transferred to a fresh glass vessel, and the remaining material was re-extracted with 600 µL chloroform, 300 µL methanol with BHT, and 300 µL water. The sample was vortexed for 30 sec, followed by incubation at -20 °C for 1 h. The mixture was then centrifuged in the same manner as before and the bottom layers were combined. The extracted sample was dried completely using a SpeedVac concentrator (Thermo Fisher Scientific Inc.). Reconstitution was achieved using 200 µL methanol with 1% chloroform.

***Metabolite preparation***

LFF (100 µL) and GOF (100 mg) were extracted using 1 mL ice-cold methanol. The samples were vortexed for 30 sec, followed by centrifugation for 5 min at 1500 rpm. The supernatant was dried completely in a SpeedVac concentrator (Thermo Fisher Scientific Inc.). Extracts were reconstituted using 15 min of sonication in 200 µL methanol.

***Protein preparation***

Protein extraction was performed using 5 different extraction methods. For each extraction method, 100 µL LFF and 100 mg GOF were used. Ice-cold extraction solvents (methanol, acetonitrile, and acetone) were added separately to each sample to precipitate the proteins. Following precipitation, the samples were centrifuged at 12,000 × *g* for 10 min. The supernatant was discarded. The protein pellet was air dried and reconstituted with 0.2% surfactant enhancer (Promega Corporation, Madison, WI, USA). Extraction was also performed using lysis buffer (SDS lysis buffer and NP 40 lysis buffer). Lysis buffer was added separately to each sample and vortexed for 1 h.

The extracted proteins were analyzed on a Qubit 3 Fluorometer (Thermo Fisher Scientific Inc.) to determine the total protein concentration. Sample aliquot equivalent to 10 µg of total protein was loaded onto a 12.5% tris-HCl Criterion precast gel (Bio-Rad Laboratories, Hercules, CA, USA) at 170 V for 15 min. The gel was stained with GelCode blue and bands were excised. Each band was then reduced with dithiothreitol and then alkylated with iodoacetamide. The gel bands were then washed and dehydrated with acetonitrile before performing enzymatic digestion. Trypsin digestion was performed at 50 °C for 1 h. The digestion was halted by addition of trifluoroacetic acid (TFA). The peptides were then subjected to LC-MS/MS.

***Liquid chromatography***

***Lipids.*** The lipid extracts (5 μL) were injected into a Thermo Fisher Scientific Acclaim PepMap RSLC C18 (300 µm × 15 cm, 2 µm, 100 Å pore size) with C18 pre-column (3 mm × 2 cm, 75 µm, 100 Å pore size) of the same material using a Thermo Fisher Scientific UltiMate 3000 RSLCnano system equipped with degasser, pump, column compartment, and autosampler. The column was maintained at a constant 40 °C during the analysis and the autosampler was maintained at 4°C.

Mobile phases of (A) 60/40% acetonitrile/water and (B) 90/8/2% isopropanol/ acetonitrile/water, both containing 10 mM ammonium formate and 0.1% formic acid, were used for the gradient pump at a flow rate of 5 µL/min. Sample injections were loaded onto the pre-column and washed for 5 min in 98% water with 10 mM ammonium formate and 0.1% formic acid and 2% acetonitrile with 0.1% formic acid at a 25 µL/min flow rate. After this time, the switching valve was activated to initiate column separation. The gradient pump operated at 50% B for 5 min before ramping to 75% B at 50 min and 98% B at 70 min. The column was held at 98% B for 20 min, before returning to 50% B at 95 min for column re-equilibration.

***Metabolites.*** The metabolite extracts (5 μL) were injected into a separate Thermo Fisher Scientific Acclaim PepMap RSLC C18 (300 µm × 15 cm, 2 µm, 100 Å pore size) column. Pre-column and LC system parameters were the same as for the lipid analysis.

Mobile phases of (A) water with 10 mM ammonium formate and 0.1% formic acid and (B) acetonitrile with 0.1% formic acid were used for both the gradient and loading pumps. Sample injections were loaded onto the pre-column and washed for 5 min in 2% B at a 25 µL/min flow rate. After this time, the switching valve was activated to initiate column separation. The gradient pump operated at 2% B for 5 min before ramping to 98% B at 60 min and holding for 10 min. Thereafter, the mobile phases were returned to starting conditions at 75 min, and the column was re-equilibrated before the next injection.

***Proteins***. Nano-liquid chromatography was performed using an UltiMate 3000 RSLCnano system from Thermo Fisher Scientific. The mobile phase A was water containing 0.1% formic acid and the mobile phase B was acetonitrile with 0.1% formic acid. The extracted peptides were injected on to a Thermo Scientific Acclaim Trap Cartridge (C18 column, 75 µm ID, 2 cm length, 3 mm, 100 Å pore size) at a 5 µL/min flow rate. This was held for 10 min and washed with 2% B to desalt and concentrate the peptides. The injector port was programmed to switch to inject and the peptides were eluted off the trap onto the nanocolumn (Thermo Fisher Scientific, C18, 75 mm ID, 25 cm length, 3 mm, 100 Å pore size) at a flow rate of 300 nL/min using the following gradient: 2% B, 0–10 min; 2–7% B, 10–20 min; 7–45% B, 20–240 min; 45–80% B, 240–250 min; 80% B, 250–255 min; 80–2% B, 255–256 min; 2% B, 256–260 min. The column temperature was maintained at 35 ^o^C. The total run time was 260 min.

***Mass spectrometry***

***Lipids.*** Lipidomic MS analysis was performed on an Impact II QqTOF mass spectrometer (Bruker Daltonics, Billerica, MA, USA) using Apollo electrospray ionization (ESI) operated in positive mode. The MS was operated at a capillary voltage of 4.5 kV, nebulizer of 0.3 bar, and nitrogen drying gas flow rate of 4.0 L/min and temperature at 200 °C. The instrument was programmed for data dependent acquisition (DDA) for collision induced dissociation (CID) using a nitrogen collision gas partner. DDA was selected for singly and doubly charged ions in the mass range *m/z* 500-1500 using an *m/z* 2 mass window and collision energies around 25 and 30 eV, depending on the programmed mass bracket. Active exclusion was employed to exclude an ion after a single spectrum for 3 min unless the current-to-previous intensity ratio was greater than or equal to 2.0.

A pooled QC sample using 2 µL/sample in 100 µL was acquired before and after the lipid samples to monitor the instrument operation during sample acquisition. A plug of sodium formate was acquired prior to each sample to ensure mass accuracy during the analysis.

***Metabolites.*** Metabolomic MS analysis was performed on the same Bruker QqTOF-MS instrument using the same MS source and parameters as the lipidomic analysis. The metabolomics MS was also analyzed in positive mode. The instrument was programmed for DDA using CID with a nitrogen collision gas partner. DDA was selected for singly charged ions in the mass range *m/z* 50-1500 using an *m/z* 3 mass window and collision energies around 20 and 35 eV, depending on the programmed mass bracket. Active exclusion was employed to exclude an ion after a single spectrum for 1 min unless the current-to-previous intensity ratio was greater than or equal to 2.0.

A freshly prepared plasma metabolomics (pooled normal human plasma K3 EDTA; Innovative Research Inc., Novi, MI, USA) QC sample was acquired before and after the metabolite samples to monitor the instrument operation during sample acquisition. A plug of sodium formate was acquired prior to each sample to ensure mass accuracy during the analysis.

***Proteins.*** Proteomics MS analysis was performed using a Thermo Scientific Q Exactive HF Orbitrap mass spectrometer equipped with an EASY Spray nanospray source (Thermo Fisher Scientific Inc.) in positive ion mode. The EASY Spray source was operated with a spray voltage of 1.5 kV and a capillary temperature of 200 ^o^C. The scan sequence of the mass spectrometer was based on the original TopTen™ method. The analysis was programmed for a full scan recorded between 375–1575 Da at 60,000 resolution and a MS/MS scan at resolution 15,000 to generate product ion spectra to determine amino acid sequence in consecutive instrument scans of the fifteen most abundant peaks in the spectrum. The AGC Target ion number was set at 3e6 ions for full scan and 2e5 ions for MS^2^ mode. Maximum ion injection time was set at 50 msec for full scan and 55 msec for MS^2^ mode. Micro scan number was set at 1 for both full scan and MS^2^ scan. The HCD fragmentation energy (N)CE/stepped NCE was set to 28. Singly charged ions were excluded from MS^2^. Dynamic exclusion was enabled with a repeat count of 1 within 30 sec and to exclude isotopes. A siloxane background peak at 445.12003 was used as the internal lock mass.

HeLa protein digest standard is used to evaluate the integrity and the performance of the columns and mass spectrometer. If the number of protein IDs from the HeLa standard falls below 2700, the instrument is cleaned, and new columns installed.

***Bioinformatics and data processing***

***Lipids and Metabolites.*** Spectral analysis was performed in Compass (version 5.1; Bruker Daltonics, Billerica, MA, USA). Bioinformatics was performed in MetaboScape (version 4.0; Bruker Daltonics, Inc.) and SimLipid (version 6.05; PREMIER Biosoft, San Francisco, CA, USA). A signal threshold of 1,000 counts for the LFF and GOF was chosen for the metabolomics data. For the LFF and GOF lipidomic data, the signal threshold was set at 20,000 and 200,000 counts, respectively.

Lipid and metabolite annotations were performed using spectral libraries from SimLipid, HMBD metabolites, and MassBank of North America (MoNA) libraries applying an *m/z* match of 3.0–5.0, mSigma match of 20–50, and MSMS score of 900–500. Metabolomic data were also searched using an analyte list (MS1 mass matching) from Phenol-Explorer (version 3.6) [1]. After library annotation, SmartFormula annotation was performed using common element ratio filters for CHNOPS compounds with *m/z* matching of 1.0–5.0 ppm and mSigma matching of 20–50. Lipid category and class determination was determined using only SimLipid spectral annotations. Metabolomic compound classifications were prepared from all spectral and analyte annotations and determined with the assistance of PubChem classifications.

***Proteins.*** MS/MS data were analyzed using Proteome Discoverer software (version 2.4; Thermo Fisher Scientific Inc.). SEQUEST HT was used as the database search algorithm. SEQUEST (XCorr Only) was set up to search SwissProt TaxID=9606_and _subtaxonomies (v2018-02-28 42253 entries) assuming the digestion enzyme trypsin. SEQUEST (XCorr Only) was searched with a fragment ion mass tolerance of 0.020 Da and a precursor ion tolerance of 10.0 ppm. Carbamidomethylation of cysteine was specified in SEQUEST (XCorr Only) as a fixed modification. Deamidation of asparagine and oxidation of methionine were specified in SEQUEST (XCorr Only) as variable modifications.

**References**

1. Neveu V, Perez-Jiménez J, Vos F, Crespy V, du Chaffaut L, Mennen L, Knox C, Eisner R, Cruz J, Wishart D, Scalbert A (2010) Phenol-Explorer: an online comprehensive database on polyphenol contents in foods. Database 2010 bap024, <https://doi.org/10.1093/database/bap024>

**Table S1**

Basic characteristics of the two organic fertilizer products analyzed in this study.

| Fertilizer product | Total N  (%) | Total C  (%) | C/N ratio | Organic N  (%) | pH |
| --- | --- | --- | --- | --- | --- |
| Liquid fish fertilizer | 4.97 | 14.12 | 2.84:1 | 3.90 | 3.5 |
| Granular organic fertilizer | 10.09 | 36.22 | 3.59:1 | 9.73 | 6.0 |

The fertilizer sample analysis was conducted by Waters Agricultural Laboratories, Inc. (Camilla, GA, USA).

**Table S2**

List of metabolites annotated from the LC-MS/MS results of liquid fish fertilizer and granular organic fertilizer. Annotations were made through both product spectrum (i.e., spectral) matching and mass (i.e., analyte) matching.

| RT | m/z | Name | Formula | Ion abundance  (Liquid fish fertilizer) | Ion abundance  (Granular organic fertilizer) |
| --- | --- | --- | --- | --- | --- |
| 33.82 | 323.2582 | _-_-Spongiane-15,16-diol | C_20_H_34_O_3_ | 27889 | 2839 |
| 30.23 | 321.2427 | _-Beyerol | C_20_H_32_O_3_ | 42052 | 297 |
| 26.14 | 351.2530 | _NCGC00160223-01_3,16,17-trihydroxy-17-acetyl-androstane_ | C_21_H_34_O_4_ | 35678 | 0 |
| 26.58 | 351.2539 | _NCGC00160223-01_3,16,17-trihydroxy-17-acetyl-androstane_^a^ | C_21_H_34_O_4_ | 385828 | 5120 |
| 30.51 | 320.2586 | 1,4a-dimethyl-6-methylidene-5-_2E_-3-methylpenta-2,4-dienyl_-3,4,5,7,8,8a-hexahydro-2H-naphthalene-1-carboxylic acid | C_20_H_30_O_2_ | 50752 | 7102 |
| 29.90 | 311.2369 | 14_-apo-beta-carotenal | C_22_H_30_O | 292863 | 21392 |
| 43.86 | 388.3572 | 25-Azacholesterol | C_26_H_45_NO | 230016 | 3262 |
| 40.16 | 386.3415 | 25-azavitamin D3_25-azacholecalciferol | C_26_H_43_NO | 351054 | 6734 |
| 22.11 | 357.2787 | 3β-Hydroxy-5-cholenoic acid | C_24_H_38_O_3_ | 73182 | 0 |
| 17.59 | 424.3060 | 3-Oxocholic acid | C_24_H_38_O_5_ | 70891 | 0 |
| 23.54 | 282.2789 | 5-_1-Hydroxypropan-2-yl_isolongifolane | C_18_H_32_O | 0 | 4127 |
| 37.84 | 207.1017 | Acetyl eugenol | C_12_H_14_O_3_ | 20093 | 351 |
| 41.94 | 207.1016 | Acetyl eugenol (MS1) | C_12_H_14_O_3_ | 5580 | 111 |
| 31.51 | 207.1020 | Acetyl eugenol (MS1) | C_12_H_14_O_3_ | 1463 | 45 |
| 36.20 | 207.1017 | Acetyl eugenol (MS1)^a^ | C_12_H_14_O_3_ | 9875 | 327 |
| 23.56 | 131.0858 | Anethole (MS1) | C_10_H_12_O | 4441 | 0 |
| 26.54 | 149.0964 | Anethole (MS1) | C_10_H_12_O | 6389 | 0 |
| 27.81 | 149.0964 | Anethole (MS1) | C_10_H_12_O | 5591 | 0 |
| 29.90 | 149.0965 | Anethole (MS1) | C_10_H_12_O | 15312 | 1313 |
| 28.22 | 149.0964 | Anethole (MS1)^a^ | C_10_H_12_O | 25251 | 2218 |
| 45.14 | 151.0757 | 3-Methoxyacetophenone (MS1) | C_9_H_10_O_2_ | 1268 | 0 |
| 17.37 | 139.0759 | 4-Ethylcatechol (MS1) | C_8_H_10_O_2_ | 3204 | 0 |
| 41.43 | 321.2783 | 5-Pentadecylresorcinol (MS1)^a^ | C_21_H_36_O_2_ | 2013 | 135 |
| 43.41 | 321.2779 | 5-Pentadecylresorcinol (MS1) | C_21_H_36_O_2_ | 1006 | 0 |
| 18.28 | 274.2741 | C16 Sphinganine | C_16_H_35_NO_2_ | 544 | 2790 |
| 17.87 | 451.3300 | C17 Sphingosine-1-phosphocholine | C_22_H_47_N_2_O_5_P | 4347 | 0 |
| 23.54 | 300.2893 | C18_Sphingosine | C_18_H_37_NO_2_ | 0 | 4385 |
| 15.05 | 308.2218 | Capsaicin | C_18_H_29_NO_3_ | 0 | 9817 |
| 20.77 | 333.2061 | Carnosic acid (MS1) | C_20_H_28_O_4_ | 6261 | 0 |
| 28.21 | 151.1120 | Carvacrol (MS1) | C_10_H_14_O | 4852 | 508 |
| 18.98 | 373.2737 | Cholacalcioic acid _ 25,26,27-trinorvitamin D3 24-carboxylic acid _ 25,26,27-trinorcholecalciferol 24-carboxylic acid | C_24_H_36_O_3_ | 105627 | 1391 |
| 16.42 | 426.3219 | Cholic acid | C_24_H_40_O_5_ | 10551 | 0 |
| 18.98 | 426.3214 | Cholic acid^a^ | C_24_H_40_O_5_ | 254046 | 3385 |
| 24.00 | 301.2163 | Dehydroabietic acid ^a^ | C_20_H_28_O_2_ | 22921 | 0 |
| 24.42 | 301.2162 | Dehydroabietic acid | C_20_H_28_O_2_ | 12789 | 0 |
| 30.01 | 300.2897 | DErySphingosine | C_18_H_37_NO_2_ | 33914 | 2462 |
| 41.37 | 279.1591 | Dibutyl phthalate | C_16_H_22_O_4_ | 8807 | 0 |
| 41.37 | 391.2841 | Dioctylphthalate | C_24_H_38_O_4_ | 24965 | 824 |
| 42.44 | 340.3572 | Docosanamide | C_22_H_45_NO | 35036 | 502 |
| 41.87 | 338.3416 | Erucamide | C_22_H_43_NO | 40960 | 4984 |
| 34.64 | 348.2898 | Ethyl icosapentate | C_22_H_34_O_2_ | 34057 | 1231 |
| 17.37 | 139.0759 | Ethylcatechol (MS1) | C_8_H_10_O_2_ | 3204 | 0 |
| 19.20 | 277.1798 | Gingerol | C_17_H_26_O_4_ | 88189 | 101 |
| 32.26 | 346.2741 | Himbacine | C_22_H_35_NO_2_ | 0 | 26228 |
| 37.08 | 346.3100 | Macamide | C_23_H_39_NO | 34657 | 486 |
| 33.67 | 357.2999 | Monoolein | C_21_H_40_O_4_ | 0 | 62054 |
| 19.99 | 272.2586 | Myristoyl-EA | C_16_H_33_NO_2_ | 21165 | 0 |
| 37.19 | 374.3415 | N-amyl arachidohoyl amine | C_25_H_43_NO | 39140 | 500 |
| 39.30 | 360.3259 | N-butyl arachidonoyl amine | C_24_H_41_NO | 716771 | 15322 |
| 32.81 | 331.2842 | NCGC00186665-03_2,3-dihydroxypropyl hexadecanoate | C_19_H_38_O_4_ | 1161326 | 134891 |
| 35.84 | 404.3158 | NCGC00380823-01_2-_14-methylpentadecanoylamino_-3-phenylpropanoic acid | C_25_H_41_NO_3_ | 22797 | 0 |
| 26.33 | 303.2317 | NCGC00385046-01_C20H30O2_Pimara-9_11_,15-dien-18-oic acid | C_20_H_30_O_2_ | 3789 | 0 |
| 30.49 | 303.2319 | NCGC00385046-01_C20H30O2_Pimara-9_11_,15-dien-18-oic acid^a^ | C_20_H_30_O_2_ | 22173 | 2620 |
| 20.55 | 391.2844 | Nutriacholic acid | C_24_H_38_O_4_ | 97573 | 0 |
| 23.33 | 300.2896 | Palmitoyl-EA | C_18_H_37_NO_2_ | 99063 | 0 |
| 15.70 | 193.0869 | *p*-Coumaric acid ethyl ester | C_11_H_12_O_3_ | 0 | 64 |
| 40.83 | 193.0859 | *p*-Coumaric acid ethyl ester (MS1) | C_11_H_12_O_3_ | 4452 | 124 |
| 36.60 | 193.0861 | *p*-Coumaric acid ethyl ester (MS1) | C_11_H_12_O_3_ | 5680 | 425 |
| 35.01 | 193.0861 | *p*-Coumaric acid ethyl ester (MS1)^a^ | C_11_H_12_O_3_ | 21510 | 0 |
| 22.55 | 318.3002 | Phytosphingosine | C_18_H_39_NO_3_ | 25672 | 614 |
| 40.04 | 336.3260 | Pipericine | C_22_H_41_NO | 7758 | 1978 |
| 28.67 | 315.2319 | Progesterone | C_21_H_30_O_2_ | 29314 | 1221 |
| 31.30 | 419.2789 | Simvastatin | C_25_H_38_O_5_ | 67718 | 2400 |
| 19.49 | 467.3607 | Sphinganine-1-phosphocholine | C_23_H_51_N_2_O_5_P | 26776 | 817 |
| 18.82 | 465.3453 | Sphingosine-1-phosphocholine | C_23_H_49_N_2_O_5_P | 151508 | 647 |
| 36.74 | 284.2949 | Stearamide | C_18_H_37_NO | 139422 | 0 |
| 34.56 | 328.3212 | Stearoyl-EA | C_20_H_41_NO_2_ | 0 | 1399 |
| 38.40 | 478.3542 | Stoloniferone G | C_28_H_44_O_5_ | 21741 | 406 |
| 16.03 | 516.2995 | Taurocholic acid | C_26_H_45_NO_7_S | 72185 | 725 |
| 18.04 | 517.3310 | Taurodeoxycholic acid | C_26_H_45_NO_6_S | 52221 | 0 |

RT: Retention time (min).

(MS1): Annotations using the analyte matching approach.

^a^ Representative annotation from several similarly annotated features (unique RT, m/z ion pairs). Representative annotations were chosen based on ion abundance.
